# Biography of the Late Dr. Denman

**Published:** 1816-01

**Authors:** 


### BIOGRAPHY OF THE LATE DR. DENMAN.

DR. THOMAS DENMAN was born on the 27th of June, 1733, at Bakewell, in the county of Derby, and was the second son of a respectable apothecary in that town, where he was educated at the grammar school. His father died in the year 1752, and he for some time assisted his elder brother, who succeeded to the business; but in his 21st year he came, with the slender patrimony of 75l., to London, where he attended St. George's Hospital several months, and two courses of lectures on anatomy. He then procured an appointment as surgeon's mate in the navy, and, being made surgeon in 1757, through the interest of the Duchess Dowager of Devonshire, he, after a cruise of seventeen months off the coast of Africa, was appointed to the Edgar, a new 60-gun ship, commanded by Captain, afterwards Admiral, Drake, with whom he continued till, on the conclusion of peace in 1763, he left the navy. During his nine years' service, he formed many valuable friendships, which he preserved through life, particularly with the amiable and excellent officer whose name has been mentioned; mentioned: his mind was enlarged by general reading, and by visiting various parts of the world; and, having been present at most of the important naval operations of that war, he materially improved his medical skill and knowledge. At the siege of the Havannah (as on a former occasion, when he assisted in the hospital at Gibraltar, then containing not less than 1100 patients), he contracted a dangerous illness, from too close an attendance on the sick and wounded. On returning to his native country, he continued, as before, to pursue his professional studies in London, and attended the Lectures on Midwifery then given by Dr. Smellie; but, obtaining, in 1764, a diploma from the University of Aberdeen, he endeavoured to establish himself at Winchester. This attempt proving unsuccessful, he again took up his residence in the metropolis, where his prospects were so little flattering, that he actually made an effort to resume the situation of a surgeon in the navy, but was unable to procure a warrant. Under these circumstances, the surgeoncy of one of the royal yachts, which he owed to the influence of Lord John Cavendish, and the friendly recommendation of Captain Drake, and which brought a salary of 70l. a year, without materially affecting his London practice, afforded an important addition to his small income. About the same period he became more generally known by the publication of some medical tracts, and commenced those Lectures in Midwifery, in conjunction with the late Dr. Osborne, which they continued to deliver for fifteen years with great reputation. In the same year he was appointed joint physician and man-midwife to the Middlesex Hospital. With these aids, and by a rare union of patience, industry, and frugality, with an ardent temper, an independant spirit, an honest ambition, and singular zeal in his profession, he was enabled to emerge, by slow degrees, from obscurity to the extensive practice and eminent character which he so long enjoyed. He was appointed licentiate in midwifery by the College of Physicians in 1783, and six years after was elected an honorary member of the Edinburgh Royal Society. Dr. Denman's progress towards the first practice was, however, the more slow, because Dr. Hunter had long been in possession of the public confidence, and because Dr. Ford was at the same time in extensive business. But, when he had reached the summit of his branch of the profession, Dr. Denman kept his station with a firmness of which there have been few examples. This arose from his full and well grounded knowledge, from his strong natural sagacity, from the most perfect uprightness of conduct, and from the benevolence of his character. In 1791, Dr. Denman purchased a small country-house at Feltham, near Hounslow, and in some measure withdrew from business; but he never quitted it entirely, and, to the latest period of his life, preserving the unabated confidence of the public, he may be truly said to have possessed that of the members of his own profession even in a greater degree. At the very advanced period to which he lived, he retained, to a wonderful extent, the vigour of his body, and, quite unimpaired, the vigour of his mind; but what is is still more singular, he retained also, without decay, all the kindly affections of his nature, with all the cheerful animation of youth, and exercised an active benevolence to the very last.

In the year 1770, Dr. Denman married Elizabeth, the youngest daughter of Alexander Brodie, a respectable linen draper in London,—a companion well suited to him from the uprightness of her mind, the soundness of her religious principles, and the benevolence of her character. They had a son and two daughters: his wife and all his children survive him.

It is believed that Dr. Denman's earliest publication was a treatise on Puerperal Fever, which appeared about the year 1770, and was soon followed by a Letter to Dr. Huck on the Construction and Use of Vapour Baths. From this period, he was constantly in the habit of printing short tracts on subjects connected with his branch of the profession, which have been, from time to time, incorporated in the several editions of his Aphorisms on the Application and Use of the Forceps and Vectis, and of his Introduction to the Practice of Midwifery, both of which works are too well known in the medieal world to reqnire, in this place, particular notice. They have each gone through five editions; and a sixth of the latter was nearly prepared at the time of the author's death. One of the former editions of it was in quarto, and was accompanied by fifteen engravings, made at a very considerable expence, on the generation and parturition of the Human Species, and of Animals. The preface, independantly of its professional merits, exhibits much general knowledge and information, and has been highly estimated by good judges as a literary composition. The work has been translated into French, and in that language also is believed to have passed through more than one edition.

Several papers, written by Dr. Denman, will be found in the 5th, 7th, and 11th volumes of the London Medical Journal, conducted by Dr. Foart Simmons. After that publication was discontinued, he became a frequent contributor to the London Medical and Physical Journal. He was among the first to recognize the important discovery of his friend and pupil Dr. Jenner; and having satisfied his own mind of its truth and utility, he did not hesitate to announce his conviction, and to support it by some striking facts, the result of his own inquiries, at a time when vaccination had made a comparatively small progress in public opinion. Two letters by him on this subject appeared in the 3d and 4th volumes of the work last mentioned. Communications more immediately connected with his own pursuits were published in the 2d and 14th volumes. In the 27th he wrote a paper on the Structure of Cancerous Parts, and a Description of a curious Case of Polypus; and in the 34th another paper on Cancer.

He published in 1809, in the 3d volume of the Transactions of a Society for the Improvement of Medical and Surgical Knowledge, a very valuable paper on Excrescences of the Womb; and, in 1810, a pamphlet consisting of treatises on the Rupture of the Uterus, on the Snuffles in Infants, and on Mania Lactea.

a very

Dr. Denman always felt a strong desire to discover a remedy for cancer, and deemed it almost criminal in medical men to despair of an object so interesting to humanity. To pronounce that dreadful disease incurable, he thought was in some degree to make it so; and his mind was continually on the watch for information on the subject. In 1801, indeed, he was instrumental in forming a charity for the exclusive relief of persons afflicted with cancer, and recommended it by a circular paper, which may be considered as an admirable specimen of the method to be pursued in conducting medical enquiries. Some account of that establishment, which was given up after a trial of a few years, is contained in a pamphlet "on the Cure of Cancer," published by him in 1810: in the following year he wrote observations in this Journal on the review of his pamphlet, which it had given. When Mr. Young made public his mode of treating cancer, Dr. Denman was disposed to flatter himself that this great end was on the eve of being accomplished; he gratuitously attended several patients under the care of that gentleman, in order to become acquainted with his practice; and, having thus ascertained that it was both innocent and beneficial, and believing that it held forth a fair promise of the most important consequences, he introduced it to the public attention by a letter which was inserted in the October number of this Journal. With the same view, he prepared for the press a second edition of his pamphlet on the subject, subjoining a full statement of Mr. Young's system, and his own remarks upon it.

Good sense and practical utility are the leading features in all Dr. D.'s compositions. They not only explain with clearness what had been before discovered, and add the sanction of the author's experience, where it accords with former opinions, but they perform the still more valuable service of correcting such opinions when found to be erroneous, and of enriching the stock of previous knowledge with original observations. It may be stated in particular that the formidable disease in children called the malignant snuffles, was very little known till it was described by Dr. Denman, who had also the good fortune to point out a mode of treating it, which is generally successful. The evolution of the child, in certain circumstances, by the action of the uterus, is a most curious and important fact, first discovered by him. He was the first also who recommended, in cases of retroverted uterus, the frequent emptying of the urinary bladder by a catheter, as all that was necessary for the restoration of the womb to its proper position. The excitement of premature labour, for the preservation both of the mother and the child, in certain cases of deformed pelvis, is a most important operation suggested by him, which has been successful in almost every instance where it has been adopted. In convulsions preceding labour, particularly where the pulse is slow, he recommended copious bleeding, instead of nervous medicines, and advised that there should be no artificial delivery, until the head of the child shall have passed through the os uteri. These are examples of important practical improvement, which will preserve Dr. Denman's name in remembrance, and give him a just claim to the gratitude of posterity.

This enumeration of his works is most probably incomplete; but those which have been mentioned will sufficiently prove that the author was constantly employed in endeavouring to improve the medical art, and diminish the extent of human suffering. It is apprehended that they also exhibit an understanding peculiarly gifted with all the faculties, and regulated by all the dispositions, that are most favourable to the investigation of truth. And, when it is remembered that they were all composed in the midst of unceasing professional engagements, always severe, and frequently both harassing and afflicting, they will be allowed to evince an unwearied activity and perseverance in the pursuit of benevolent and useful objects, which are excelled only by the motives that prompted their exertion.

*Dec.* 22, 1815.

For the above we are indebted to a source the faithfulness of which cannot be questioned.—Edit.

